# OLDER WOMEN'S EXPERIENCES OF AGING: THE PHYSICAL, THE PSYCHOLOGICAL, AND THE SOCIAL

**DOI:** 10.1093/geroni/igz038.777

**Published:** 2019-11-08

**Authors:** Nicky J Newton

**Affiliations:** Wilfrid Laurier University, Waterloo, Canada

## Abstract

According to the life course perspective (Settersten, 2003), major life transitions are embedded in contexts shaped by personal history and social circumstances “as natural as the changing seasons” (Miller, 2010, p.663). Aging itself is perhaps the epitome of all transitions: a relatively measured movement through a series of situations, conditions, and social roles (Hettich, 2010); a transition that particularly lends itself to a life course approach. In this qualitative interview study, 37 women (Mage = 72.27) responded to questions regarding their experiences of the physical, psychological and social aspects of aging. While themes of inevitability and physical health were evident, the highly-personalized nature of aging was also underscored through individual themes of invisibility, freedom from expectations, fear of cognitive decline, and the quality and maintenance of friendships. Similarities and differences in women’s experience of aging are compared; the need to contextualize aging within the life course is discussed.

